# Comparison between the Interferon *γ* Release Assay—QuantiFERON Gold Plus (QFT-Plus)—and Tuberculin Skin Test (TST) in the Detection of Tuberculosis Infection in Immunocompromised Children

**DOI:** 10.1155/2020/7159485

**Published:** 2020-05-10

**Authors:** Cory Primaturia, Lelani Reniarti, Heda M. N. Nataprawira

**Affiliations:** Department of Child Health, Faculty of Medicine Universitas Padjadjaran/Dr. Hasan Sadikin General Hospital, Jl. Pasteur No. 38 Bandung 40161, Indonesia

## Abstract

**Background:**

Immunocompromised patients are at a higher risk of having latent tuberculosis infection (LTBI). QuantiFERON-TB Gold Plus (QFT-Plus) has been proven to perform effectively in LTBI detection among immunocompromised adults and can overcome the limitations of the tuberculin skin test (TST). However, the role of QFT-Plus in detecting LTBI in immunocompromised paediatric patients has not been well established. Therefore, the aim of this study was to assess the test agreement between QFT-Plus and the TST in LTBI detection among immunocompromised children.

**Method:**

In this cross-sectional study, we enrolled immunocompromised paediatric patients, aged between 5 and 18 years, who were treated with corticosteroids and/or chemotherapy from June to November 2019. We categorized them into three groups based on the following diseases: hematologic malignancies and nephrological and immunological diseases. We recorded the patient characteristics and QFT-Plus and TST results, in which the positive result of the TST was induration ≥ 5 mm. Within the same group, comparisons between the two tests were performed using the McNemar test, and results were statistically significant for *p* values of <0.05. The kappa index was used to assess the agreement between the two test results.

**Results:**

Among 71 patients (median age: 11.8 years) who underwent TST and QFT-Plus testing, 52% were females, and 69% had a normal nutritional status. Chemotherapy was the most common treatment modality for hematologic malignancy compared to other immunosuppressive treatments. The total number of patients with positive QFT-Plus and TST results was 11/71 (15.5%) and 4/71 (5.6%), respectively, among whom 3/11 patients had positive results in both tests, and one patient with positive TST results exhibited a discrepancy, as this was not followed by positive QFT-Plus results. QFT-Plus generated more positive results than the TST in immunocompromised children (McNemar, *p* = 0.039 (*p* < 0.05)). The diagnostic agreement between the tests was fair (*K* = 0.345, 95% CI: 0.05–0.745).

**Conclusion:**

QFT-Plus detected LTBI more effectively than the TST in immunocompromised children.

## 1. Introduction

Tuberculosis (TB) is still one of the health global problems in the world, including the children population. Based on the Global Tuberculosis Report 2019, Indonesian children with new and relapse TB cases are about 11% of the total TB incidence as estimated in 2018. However, there are many cases of TB that remain unrecognized, incorrectly diagnosed, and unreported. The World Health Organization (WHO) reported that Indonesia is a country with the third highest TB incidence, after India and China [1]. Currently, the TB eradication program is not only focused on treatment but also on prevention by conducting contact investigations and diagnosing latent tuberculosis infection (LTBI) so that the infection does not develop into TB disease by giving isoniazid (INH) prophylaxis treatment [1, 2]. Latent tuberculosis infection (LTBI) is a condition associated with a persistent immune response to the *Mycobacterium tuberculosis* (Mtb) antigen that produces neither any clinical manifestations nor X-ray findings indicating the presence of the disease [3]. LTBI treatment coverage reported low in Indonesia, especially child contacts < 5 years old is about 11% [4]. The studies on LTBI of the immunocompromised population are still limited, while the prevalence of LTBI has been estimated to affect almost one-third (2.3 billion) of the global population [5]. Screening for LTBI is a crucial component of the World Health Organization's (WHO) End TB Strategy [1, 5].

Immunocompromised adult patients with solid organ transplant (SOT), hematopoietic stem cell transplant (HSCT), and hematologic malignancy were reported to have a higher risk of progression to active TB, with a risk between 20 and 74 times greater than the general population [6–10]. Therefore, early detection of LTBI through screening immunocompromised patients is recommended [9, 11, 12]. The latest diagnostic method for LTBI involving the use of interferon-gamma release assays (IGRAs) was developed to replace the tuberculin skin test (TST) [9, 12–16]. The limitations of the TST, including the resulting high percentage of false-negative results in the case of immunocompromised patients, have previously been reported [12, 13, 17, 18]. The use of IGRAs is an alternative diagnostic approach for LTBI detection, and this technique has been observed to be more effective than the TST in the case of immunocompromised adult patients [14, 15, 19].

QuantiFERON-TB Gold Plus (QFT-Plus) is the latest generation of IGRAs. The QFT-Plus kit consists of two tubes of specific TB antigens (TB1 and TB2) and is able to stimulate interferon gamma (IFN-*γ*) production in the CD4 and CD8 cells on the exposure to the Mtb-specific antigen [13, 15]. The ability of QFT-Plus to detect the CD8 cell response to the Mtb antigen makes QFT-Plus better than the TST in detecting immune-mediated cell responses in an immunocompromised population with a low CD4 count [14, 20]. To the best of our knowledge, there are limited studies that have been conducted on the adult immunocompromised population, and the results of studies on immunocompromised children still remain unclear. Therefore, the aim of this study was to assess the test agreement between QFT-Plus and the TST in LTBI detection among immunocompromised children.

## 2. Materials and Methods

### 2.1. Ethical Approval

This study was approved by the ethical committee of RSUP Dr. Hasan Sadikin General Hospital, Bandung, under the identification number LB.02.01/X.6.5/122/2019, on 26 April 2019 and followed the principles outlined in the Declaration of Helsinki.

### 2.2. Study Setting

The study was conducted at the immunocompromised children's ward of RSUP Dr. Hasan Sadikin Hospital, a zonal referral hospital that caters to the West Java province, in Bandung.

### 2.3. Study Design and Population

This was an observational analytical study with a cross-sectional design, and it involved immunocompromised children who were hospitalized from June to November 2019. All of the patients were simultaneously tested using QFT-Plus (Qiagen, Hilden, Germany) and the TST after screening them based on the eligibility criteria. The patients with positive and indeterminate results by either QFT-Plus or the TST were treated isoniazid prophylaxis 10 mg/kg/day for 6 months [3]. The inclusion criteria for this study were children aged 5–18 years who were in an immunocompromised condition, including children who were undergoing chemotherapy, immunosuppressive therapy, or steroid therapy with drug doses of 1.5 mg/kg BW/day or 15 mg/day for >1 month, and those who were tested via both QFT-Plus and the TST [12, 15–17]. The exclusion criteria were as follows: patients suspected of having TB and those with a history of receiving TB therapy or of being clinically (cough ≥ 2 weeks, fever ≥ 2 weeks, body weight loss, and malaise) or microbiologically (bacteriologically positive in the rapid molecular test, GeneXpert) diagnosed with TB [2]. The characteristics of each patient were recorded (sex, age, nutritional status, history of chemotherapy, therapy duration, vaccination history, and laboratory findings, including haemoglobin, haematocrit, erythrocyte, leukocyte, thrombocyte, lymphocyte, and albumin levels). For nutritional status, we measured body mass index (BMI) for age, in which defined according to WHO child growth standard deviation (SD) or *Z* scores and were classified using the following cut-offs: for normal status (BMI for age < -2–+2 SD), moderately malnourished (BMI for age < -2 SD), and severely malnourished (BMI for age < -3 SD) [21].

### 2.4. QuantiFERON Gold Plus (QFT-Plus)

A blood sample measuring as much as 5 mL was drawn from each patient's vein and was then transferred into a lithium heparin blood tube. During the delivery process, the blood samples were stored at 2–8°C in order to maintain lymphocyte viabilities. Subsequently, the enzyme-linked immunosorbent assay (ELISA) method was used to calculate the IFN-*γ* levels in response to the exposure to the specific Mtb antigen (cocktail peptide early secretory antigen target (ESAT-6), culture filtrate protein CFP-10 (TB1) without TB7.7, and six short peptides (TB2)). The results were considered positive if the interferon concentration at ≥0.35 IU/L or ≥25% in the control tube was negative, and they were considered indeterminate if the concentration was <0.35 IU/mL or <25% with a mitogen concentration of <0.5 IU/mL [20].

### 2.5. Tuberculin Skin Test (TST)

The TST was performed using 0.1 mL of purified protein derivative (PPD RT23) that was injected intradermally into the volar surface of the hand. The PPD was stored in a cold environment (2–8°C). An induration assessment was performed 48-72 h following the injection. The results of the TST were interpreted by a paediatrician who determined them to be positive if the induration was found to be ≥5 mm [2].

### 2.6. Data Management and Statistical Analysis

Data were expressed in numbers (percentages) or median and were analysed using the IBM SPSS Statistics (version 23.0) software. The concordance between the QFT-Plus and TST assay results was assessed using kappa coefficients and was interpreted according to the Landis and Koch classification. Consequently, the Cohen's kappa test results were classified as follows: a score of <0.2 indicated slight reliability; 0.21–0.40, fair reliability; 0.41–0.60, moderate reliability; 0.61–0.80, substantial reliability; and 0.81–1, almost ideal reliability [22]. All the reported *p* values were calculated with the statistical significance set at a *p* value of less than 0.05.

## 3. Results

### 3.1. Population Characteristics

**Figure 1:**
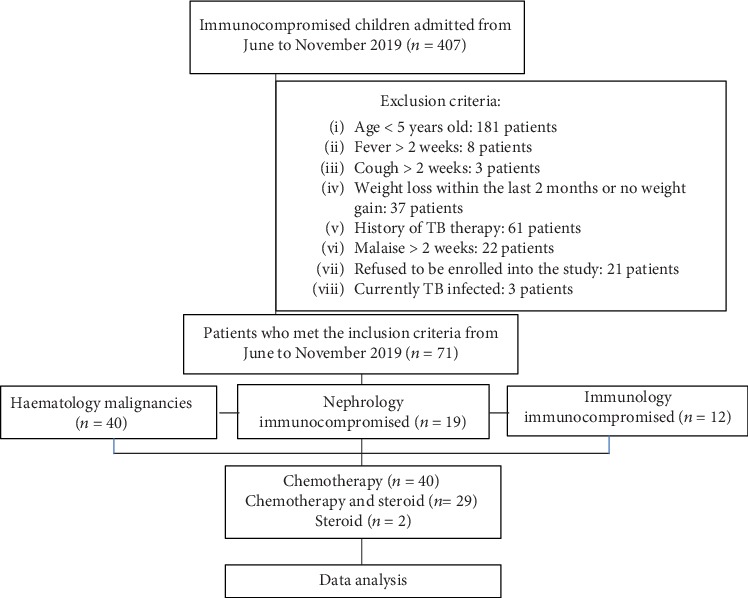
Flow diagram. TB: tuberculosis.

We prospectively included 407 consecutive immunocompromised patients who were undergoing chemotherapy and steroid treatment from July to November 2019. During this time period, there were only 71 patients who were eligible as per the study criteria. The procedure involved in patient selection is presented in Figure 1.

The types of hematological cancer in this study were acute lymphoblastic leukaemia, acute myeloblastic leukaemia, chronic lymphoblastic leukaemia, lymphoma, and solid malignant tumors (osteosarcoma, ovarian tumors, and retinoblastoma). The nephrological and immunological conditions in this study were nephrotic syndrome, steroid resistance, and systemic lupus erythematosus (SLE). The characteristics of the patients are presented in Table 1.

Among the 71 patients who had undergone QFT-Plus and the TST, the baseline clinical characteristics between the sexes were nearly equal. The median age was 11.8 years old, and most of the patients had a normal nutritional status (69.0%). Regarding the treatment that the patients underwent, 56.3% of them received chemotherapy while 40.8% received a combination of chemotherapy and steroid treatment; the duration of therapy was more than 3 months in most cases (59.2%). There were 57 (80.3%) patients who had undergone GeneXpert evaluation, and all of them generated negative rapid molecular test results. Further, 67 (94.4%) patients had Bacillus Calmette–Guérin (BCG) scars and histories of receiving BCG vaccinations. The nutritional status, history of chemotherapy, and duration of administration of immunosuppressive drugs were compared between the TST and QFT-Plus test groups as shown in Table 2.

The percentage of patients with a normal nutritional status who received positive results on the TST was 6.1% and that of patients who received positive results with QFT-Plus was 14.3%; contrarily, 10% of severely malnourished patients generated positive results on the TST and 30% exhibited positive results with QFT-Plus. Based on the therapy history, three patients (7.5%) who were treated with chemotherapy and one (2.5%) patient who was treated with chemotherapy and steroid treatment together received positive TST results; further, 26% and 15% of patients who underwent chemotherapy and steroid treatment together and chemotherapy alone, respectively, received positive QFT-Plus results, and one patient received indeterminate QFT-Plus results. Based on the duration of therapy, i.e., ≤3 months or >3 months, the percentage of the patients who generated positive results on the TST did not differ to a large extent (3.3% and 7.3%) between the two groups whereas the percentages of those with positive QFT-Plus tests were 13.3% and 17.1% in the two groups, respectively. All the immunocompromised conditions did not lead to significant differences in both the TST and QFT-Plus groups (*p* > 0.05). BCG vaccination status in these two groups statically did not show significant differences.

The laboratory examination that was performed included tests to determine the levels of haemoglobin, haematocrit, erythrocytes, leukocytes, thrombocytes, lymphocytes, and albumin (Table 3). There were no significant differences in the laboratory results between the TST and QFT-Plus test (*p* > 0.05).

In this study, the total number of patients with positive results in QFT-Plus and the TST was 11/71 (15.5%) and 4/71 (5.6%), respectively, out of whom 3/11 patients had positive results in both tests, and one patient with positive TST results exhibited a discrepancy as the positive TST results were not followed by positive QFT-Plus results. The comparison between QFT-Plus and the TST for use in LTBI diagnosis yielded statistically significant differences (*p* = 0.039), as shown in Table 4. The agreement value associated with the results of both the diagnostic approaches based on the kappa index was 0.345 (*K* < 0.4) (95% CI: 0.05–0.745), and it was subsequently classified as being fair.

## 4. Discussion

This study has demonstrated that the number of patients who exhibited positive TST results was smaller (5.6%) than that of those with positive QFT-Plus results (15.5%) associated with LTBI, with a kappa score indicating fair reliability. Compared to the results of previous studies on adult immunocompromised patients [14], our study is quite similar to those studies, with the result for IGRA test (15.1%) and TST (10.9%) with a moderate kappa score [14]. In a previous report, QFT-Plus positive results were compared to TST ones (93.7% and 73.7%, respectively) yielding higher kappa scores in an HIV-affected population [23].

Blood cancer patients have a higher case index of LTBI compared to other immunocompromised groups of patients. Blood cancer has been associated with severe immunocompromised states long before chemotherapy was developed to treat this condition [9, 10, 15, 24]. Further, blood cancer patients are associated with higher risks of developing neutropenia or leukopenia, and therefore, the risk of LTBI also increases in them [25]. This study compared results between patients who were treated with chemotherapy for ≤3 months and those who were treated for >3 months. In the QFT-Plus group, we observed that positive results were exhibited by 13.7% of the patients who were treated for ≤3 months and by 16.6% of the patients who were treated for >3 months, and these results were relatively similar to those of the TST group. We speculated that a longer duration of therapy could aggravate the immunocompromised status of a patient and increase the risk of LTBI. QFT-Plus produced more specific results than the TST because this test contains protein antigens, ESAT-6, CFP-10, and TB 7.7, that cannot be obtained via the BCG vaccination [18, 19]. We observed a discrepancy in the TST results of one of the patients who did not receive a corresponding positive QFT-Plus result. This result can be caused by the lower specificity of the TST, which is influenced by BCG vaccination.

In immunocompromised patients, false-negative results arise due to the lower production levels of cytokines, such as IFN-*γ* and tumor necrosis factor (TNF-*α*), that play roles in the immune response against Mtb. This is due to a defective T-cell response corresponding to the production of interleukin-2 (IL-2), which is caused by T-cell receptor phosphorylation that results in disturbances in ZAP-70 (Zeta-chain-associated protein kinase 70) activation, mitogen-activated protein kinase (MAPK) protein activation related to IL-2 gene transcription, and the production of protein after T-cell stimulation against the antigen [26].

Further, IGRAs have their own disadvantages, such as higher costs, the need for advanced laboratory facilities, and the need to maintain lymphocyte viability [19, 27–29]. IGRAs can also generate indeterminate results in cases in which the patient has an immunological disorder that involves low IFN-*γ* production towards a mitogen or excessive IFN-*γ* production in the control tube. Patients with immunosuppression are usually at a higher risk of receiving indeterminate results owing to the inadequacy of their body's response toward mitogens in a positive control situation [30]. Indeterminate results are also related to a child's age, and therefore, our study excluded children below 5 years old because of their immature immune response [31, 32]. In our study, we had one (1.4%) patient whose results were considered to be indeterminate and the patient was treated with INH prophylaxis.

Indeterminate results are suspected to arise due to low levels of albumin and lymphocytes [14, 29, 30, 33]. Along with the results of QFT-Plus and the TST, this study also analysed the median values of the haemoglobin, haematocrit, erythrocyte, leukocyte, thrombocyte, lymphocyte, and albumin levels; however, the results of this analysis did not reveal any significant differences (*p* > 0.05). The median value of the lymphocyte and leukocyte levels in this study was influenced by the hyperleukocytosis states of three hematological malignancy patients with the highest leukocyte count of 300000/mm^3^. The number of lymphocytes in patients with indeterminate results was reported to be lower than that of patients with determinate results (1200 cells/mL vs. 1820 cells/mL) [14]. In addition, indeterminate results are generated more frequently in immunocompromised patients owing to anaemia, lymphopenia, hypoproteinaemia, and hypoalbuminemia. Based on our study results, malnutrition did not affect the TST and IGRA results.

The percentage of patients with a normal nutritional status who received positive results on the TST was 6.1% and that of patients with positive QFT-Plus results was 17.1%, while in the severely malnourished patients the percentages of those that received positive results on the TST and QFT-Plus were 10% and 30%, respectively. The nutritional status plays a role in influencing the TST and IGRA test results, as the weight per age (<-2.63) and body mass index per age (<-2.51) lower the odds ratio of the positive TST results and increase the incidence of indeterminate IGRA results. Malnourished patients are at a higher risk of getting infected by Mtb because of their impaired immune responses; however, indeterminate results due to low mitogen response levels are also frequently reported in these patients [32]. In this study, all the malnourished patients exhibited determinate results, and only one (1.4%) patient was found to generate indeterminate results among those with a normal nutritional status.

### 4.1. Study Limitation

Further follow-ups were not conducted for patients with the possibility of having the TB disease who were administered isoniazid prophylaxis and who received positive QFT-Plus or TST results. Additionally, neither the concentration of viable lymphocytes due to improper specimen handling nor the inability of the patient's lymphocytes to generate IFN-*γ* was measured in this study.

### 4.2. Suggestion

The QTF-Plus examination needs to be routinely performed in the case of immunocompromised children in order to facilitate the proper establishment of a diagnosis protocol for LTBI so that an early intervention could accordingly be administered. It is beneficial if a further study is conducted using diagnostic test; therefore, the sensitivity and specificity of the QTF-Plus and TST can be measured in identifying LTBI progressivity to active TB disease, so that TB disease can be prevented and it will have an impact on the reduction of treatment cost.

## 5. Conclusions

The results of this study confirm that QFT-Plus® detects LTBI more effectively than the TST in immunocompromised children.

## Figures and Tables

**Table 1:** Characteristics of the patients.

Characteristics	Number (*n* = 71)	%
Sex		
Male	34	47.9
Female	37	52.1
Age (year)		
5–9	18	25.4
10–14	31	43.7
15–18	22	31.0
Median (SD): 11.8 years		
Range: 5-18		
Nutritional status		
Normal	49	69.0
Moderately malnourished	12	16.9
Severely malnourished	10	14.1
History of chemotherapy		
Chemotherapy	40	56.3
Chemotherapy+steroid	29	40.8
Steroid	2	2.8
Duration of therapy		
≤3 months	30	40.8
>3 months	41	59.2
Patient classification		
Hematology	40	56.3
Nephrology	19	26.8
Immunology	12	16.9
Rapid molecular test (GeneXpert)		
Negative	57	80.3
Not done	14	19.7
BCG scar/history of BCG vaccination		
Positive	67	94.4
Negative	4	5.6
History of contact with TB adult patient		
Positive	2	3
Negative	69	97

SD: standard deviation; BCG: Bacillus Calmette–Guérin; TB: tuberculosis.

**Table 2:** TST and QFT-Plus results based on nutritional status, chemotherapy history, therapy duration, and patient groups.

Variables assessed	TST (*n* = 71)					QFT-Plus			
	+	−	Total (*n*)	*p* value	+	Indeterminate	−	Total	*p* value
Nutritional status									
Normal	3	46	49	0.72	7	1	41	49	0.76
Moderately malnourished	0	12	12		1	0	11	12	
Severely malnourished	1	9	10		3	0	7	10	
Therapy									
Chemotherapy	4	36	40	0.19	6	1	33	40	0.11
Chemotherapy+steroid	0	29	29		5	0	24	29	
Steroid	0	2	2		0	0	2	2	
Duration of therapy									
≤3 months	2	27	29	1.0	5	0	24	29	0.67
>3 months	2	40	42		6	1	35	42	
Patient classification									
Hematological	3	37	40	0.45	6	1	33	40	0.26
Nephrological	0	19	19		1	0	18	19	
Immunological	1	11	12		4	0	8	12	
BCG status									
Positive	4	63	67	1.0	11	1	55	67	0.65
Negative	0	4	4		0	0	4	4	

TST: tuberculin skin test; QFT-Plus: QuantiFERON Gold Plus.

**Table 3:** Laboratory findings based on the TST and QFT-Plus results.

Laboratory findings	TST (median value and range)		
	Positive	Negative	*p* value
Haemoglobin (g/dL)	10.6 (9.1-12.1)	11.5 (6.2-17.2)	0.385
Haematocrit (%)	30.5 (27.2-35.0)	33.9 (17.0-52.0)	0.353
Erythrocytes (millions/m^3^)	4.1 (3.4-4.4)	4.1 (0.13-21.74)	0.768
Leukocytes (/m^3^)	4940 (3880-16040)	9690 (1070-354120)	0.601
Thrombocytes (thousand/m^3^)	411 (283-670)	320 (3-580)	0.178
Lymphocytes (%)	18 (13-47)	17.5 (1-70)	0.606
Albumin (g/dL)	3.5 (3.1-3.9)	3.3 (0.8-38)	0.513
	QFT-Plus (median value and range)		
	Positive	Negative	*p* value
Haemoglobin (g/dL)	11.2 (9.1-16.3)	11.55 (6.2-17.2)	0.952
Haematocrit (%)	32.0 (26-45)	34 (17-52)	0.634
Erythrocytes (millions/m^3^)	4.0 (3.3-5.1)	4.1 (0.13-21.74)	0.567
Leukocytes (/m^3^)	7370 (3770-19800)	9690 (1070-354120)	0.393
Thrombocytes (thousand/m^3^)	372 (199-524)	320 (3-670)	0.279
Lymphocytes (%)	15.5 (5-47)	18.5 (1-70)	0.294
Albumin (g/dL)	3.1 (1.79-3.9)	3.3 (0.8-38)	0.648

^∗^Based on the Mann-Whitney test. TST: tuberculin skin test; QFT-Plus: QuantiFERON Gold Plus.

**Table 4:** The comparison between the QFT-Plus and TST results in diagnosing LTBI.

QFT-Plus	TST		Total (%); (95% CI)
	Positive	Negative	
Positive	3	8	11 (15.5%); (8.9–25.71)
Indeterminate	0	1	1 (1.4%); (0.2–7.6)
Negative	1	58	59 (83.1%); (72.7–90.1)
Total	4	67	71
%	(5.6%)	(94.4%)	
95% CI	2.2%–3.6%	86.4%–97.8%	

*p* (McNemar test) = 0.039; *K* = 0.345 (95% CI: 0.05–0. 745) (SE 0.161). TST: tuberculin skin test; QFT-Plus: QuantiFERON Gold Plus; LTBI: latent tuberculosis infection.

## Data Availability

The data used to support the findings of this study are available from the corresponding author upon request.
